# Supplementary material for: Genetic features of antimicrobial drug-susceptible extraintestinal pathogenic Escherichia coli pandemic sequence type 95
Source: Microbiol Spectr. 2023 Dec 7;12(1):e04189-22. doi: 10.1128/spectrum.04189-22 (PMC10783064; doi:10.1128/spectrum.04189-22)
Supplement: Supplemental legends — Legends for Tables S1 to S7. [file spectrum.04189-22-s0001.docx]

**Supplementary Table 1**

Information on source type, source details, collection year, collection continent, and collection country of whole genomes of ST95 downloaded from Enterobase.

**Supplementary Table 2**

Drug resistance genes identified from the whole collection of 1749 ST95 from Resfinder.

**Supplementary Table 3**

Frequency of drug resistance among ST95 isolates registered in the Enterobase database between 1947 and 2019 and ST95 isolates from Northern California between 2013 and 2017, by year, stratified by isolation information (BSI or UTI). This illustrates the yearly distribution of drug resistance genes.

**Supplementary Table 4**

Univariate and multivariate analysis of the pangenome of 1749 ST95 whole genome sequences.

**Supplementary Table 5**

Information of 13 SNPs on core genes and 145 accessory genes associated with drug resistance.

**Supplementary Table 6**

The accession numbers of submitted ST95 whole genome sequence on DDBJ/ENA/GenBank.

**Supplementary Table 7**

NCBI accession number and the number of matching regions of ST131 whole genome sequence that carries one or more genes of 44 ST95 genes found to be associated with ST95 strains lacking drug-resistance genes.
